# miRNAs as Non-Invasive Biomarkers for Lung Cancer Diagnosis

**DOI:** 10.3390/molecules19068220

**Published:** 2014-06-17

**Authors:** Paola Ulivi, Wainer Zoli

**Affiliations:** BiosciencesLaboratory, Istituto Scientifico Romagnolo per lo Studio e la Cura dei Tumori (IRST) IRCCS, Via Maroncelli 40, 47014 Meldola (FC), Italy

**Keywords:** miRNAs, NSCLC, diagnosis, non-invasive biomarker

## Abstract

Lung cancer is a leading cause of cancer death and late diagnosis is one of the most important reasons for the high mortality rate. Circulating microRNAs (miRNAs) represent stable and reproducible markers for numerous solid tumors, including lung cancer, and have been hypothesized as non-invasive diagnostic markers. Serum, plasma or whole peripheral blood can be used as starting material, and several methodological approaches have been proposed to evaluate miRNA expression. The present review provides an in depth summary of current knowledge on circulating miRNAs in different types of biological samples used as diagnostic markers of lung cancer. We also evaluate the diagnostic accuracy of each miRNA or group of miRNAs in relation to the different housekeeping miRNAs used. Finally, the limitations and potential of miRNA analysis are discussed.

## 1. Introduction

Lung cancer represents the most common cancer worldwide, with a high mortality rate due mainly to the fact that the disease generally only becomes clinically apparent at advanced stages [1,2]. More than 75% of lung cancers are diagnosed when the disease is locally advanced or metastatic, which results in a current 5-year survival of less than 15% [1]. Several screening strategies have been proposed to reduce cancer mortality, e.g., results from the National Lung Cancer Screening Trial showed a 20% decrease in the mortality of heavy smokers who underwent annual low-dose computerized tomographic (LDCT) screening compared to an annual chest x-ray. However, high false-positive rates, elevated screening costs and potential health risks associated with LDCT highlight the need for an alternative to this diagnostic approach [1].

A non-invasive blood biomarker assay that could complement or even replace LDCT in a lung cancer screening program would have to have two potentially important advantages over other known methods, *i.e.*, it would have to be able to identify individuals at high risk of NSCLC who requirefurther investigation with spiral CT, and it would also have to be capable of discriminating between neoplastic and non-neoplastic diseases in individuals with suspicious CT-detected nodules, thus reducing the need for serial CTs or invasive biopsies. Several circulating molecular markers have been proposed, e.g., free DNA [3,4], gene methylation [5,6] and multiple marker approaches [7,8,9]. More recently, microRNAs (miRNAs) present in body fluids have been proposed as stable and reproducible biomarkers [10,11]. The aim of this review is to provide an overview of the diagnostic potential of circulating miRNAs in lung cancer, and to evaluate the feasibility of their large-scale determination in a screening program or clinical setting.

## 2. miRNAs and Mechanisms of Release into the Bloodstream

miRNAs are endogenous 19–22 nucleotide-long non-coding RNA molecules that mediate post-transcriptional gene silencing by RNA degradation or the inhibition of translation initiation [12,13,14]. miRNAs concurrently target multiple genes and a single miRNA has the potential to regulate the activity of a molecular pathway, making these molecules attractive therapeutic targets to reverse cancer phenotypes or sensitize tumors to chemotherapy [15,16]. miRNAs are also capable of providing important information on the aggressiveness of the disease and of predicting response to specific treatment [17]. Alterations in miRNA expression have been observed in various solid tumors, including hepatocellular carcinoma [18] and lung [17], breast [19], pancreatic [20], gastric [21] and prostate cancer [22], and could potentially be used for cancer diagnosis, prognosis and response to treatment [23,24].

Previous studies have shown that miRNAs are detectable in the blood [11,25,26]. Three possible mechanisms have been hypothesized for miRNA release: energy-free passive leakage of cellular miRNAs from broken cells; active secretion of miRNAs in the form of microvesicle-free miRNAs in response to different stimuli; and active release of miRNAs through microvesicles following specific stimuli [10]. The passive release of miRNAs from broken cells occurs in conditions such as tissue damage, cell apoptosis, inflammation and metastasis, all common processes in cancer [27].

With regard to the active secretion of miRNAs through cell-derived membrane-bound microvesicles, miRNAs are carried mainly by specific exosomes, *i.e.*, 50–90-nm vesicles exocytically released by numerous cell types, and by membrane-bound particles up to 1 µm in size that are produced by the shedding of the plasma membrane [28]. Membrane-bound microvesicles have been detected in various body fluids such as serum, plasma, urine, bronchoalveolar fluid and saliva, and their release may occur under both physiological and pathological conditions [29,30]. Conversely, some studies have shown that miRNAs exist in vesicle-free form associated with protein or high-density lipoprotein complexes [31,32]. In particular, Arroyo *et al.* [32] reported that the vast majority (over 90%) of circulating miRNAs are present in ribonucleoprotein complexes in a non-membrane-bound form, and that only a minority are associated with vesicles. Furthermore, Vickers *et al.* [31] observed that miRNAs in plasma are transported and delivered to the recipient cells by high density lipoproteins.

## 3. miRNA Stability

Unlike mRNA, miRNAs show high stability in different types of biological samples, *i.e.*, formalin-fixed, paraffin-embedded clinical tissue, fresh snap-frozen material, plasma or serum, and saliva [11,26,33,34]. This high stability is due to their resistance to endogenous and exogenous RNase activity, extreme temperatures, extremes of pH (pH 1 or 13), extended storage in frozen conditions, and repeated freeze-thaw cycles [26,35]. Resistance to such arduous conditions has been attributed to the above-described encapsulation and association with protein complexes [31,32,36,37,38]. In fact, it has been demonstrated that synthetic miRNAs or purified plasma miRNAs spiked into human plasma are promptly degraded [26].

## 4. miRNAs as Circulating Biomarkers

Circulating miRNAs are found in healthy individuals, and some authors have reported that miRNA deregulation can occur under different physiological or pathological conditions. Bye *et al.* [39] demonstrated differences in circulating miRNAs in proportion to aerobic fitness level and hypothesized miR-210, miR-21 and miR-222 as potential biomarkers of cardiovascular health. Moreover, increased circulating levels of miR-1, miR-423 and miR-208 have been correlated with myocardial infarction [40], heart failure [41], and myocardial damage [42], respectively. A recent review analyzed the role of miRNA expression in different non-cancerous diseases, the authors reporting that only a subset of known/identified blood-based microRNA biomarkers have specificity for a particular disease. Levels of miR-1, miR-133a, miR-133b, miR-499, miR-21, miR-208a and miR-208b were found to be higher in the blood of individuals experiencing myocardial injury such as myocardial infarction, viral myocarditis or acute coronary syndrome with respect to healthy subjects. Moreover, 24 different microRNAs were also reported as biomarkers for hepatic injury, in particular miR-122. Numerous other miRNAs have also been found in different types of diseases with a low specificity [43].

Some studies have also revealed a correlation between serum miRNAs and different virus infections, e.g., five miRNAs (miR-197, miR-629, miR-363, miR-132 and miR-122) were shown to be capable of distinguishing varicella patients from healthy controls [44]. Furthermore, a combination of four miRNAs (miR-17, miR-20a, miR-106a and miR-376c) was able to identify patients with avian-origin influenza A (H7N9) virus [45].

Several studies have observed that circulating miRNA levels are higher in cancer patients than in healthy individuals. Lawrie *et al.* were the first to report that specific miRNAs were elevated in the serum of patients with large B-cell lymphoma with respect to healthy donors [25]. In particular, they found that three miRNAs, miR-155, miR-210 and miR-21, were significantly upregulated (5.24-, 4.15- and 2.56-fold expression change, respectively) in the serum of patients with respect to healthy donors. Chen *et al.* performed genome-wide expression profiling by Solexa sequencing, observing significant differences between miRNAs in serum and in the blood cells of cancer patients, but not in healthy individuals. This would seem to indicate that specific serum miRNAs probably derive from tumor cells or tumor tissue [11]. Moreover, Mitchell *et al.*’s 2008 study on mouse xenograft models of prostate cancer revealed a clear correlation between the amount of miRNAs found in the blood and tumor growth [26]. These researchers used a mouse model to show that human miRNAs can be detected in the blood of mice after prostate cancer xenograft transplantation, and that the amount of miRNAs was correlated with the xenografted tumor mass.

Numerous miRNAs show the same pattern of alteration, *i.e.*, increased or decreased expression, in the plasma/serum and tumor tissue of patients with various tumor types. For example, the expression of miR-17-5p has been found to be increased in both the serum [46] and tumor tissue [47,48] of lung cancer patients. An elevated level of miR-155 has also been reported in tumor tissue/cells [48,49] and plasma [25] of lymphoma patients. In some cases, however, an inverse correlation has been observed between specific circulating and tissue miRNAs. For example, although Coulouarn *et al.* found a lower level of liver-specific miR-122 in liver cancer tissue [50], an increase in the serum of patients with hepatocellular carcinoma (HCC) carrying the hepatitis B virus has also been reported [51]. These findings indicate that in some instances it might be more opportune to consider secreted miRNA rather than tissue/cellular miRNA in studies on miRNA expression. Overall, the correlation between tissue and circulating miRNAs supports the hypothesis that circulating miRNAs may reflect various aspects of human physiological status and serve as fingerprints for disease diagnosis [27].

## 5. Circulating miRNAs in NSCLC

The study by Chen *et al*. [11] represents the first comprehensive analysis of miRNAs in the serum of NSCLC patients. After miRNA profiling by Solexa sequencing, the authors identified two NSCLC-specific miRNAs, miR-25 and miR-223, which were more highly expressed in the serum of NSCLC patients than in healthy donors. In the wake of these findings, several other studies analyzed the potential diagnostic role of miRNAs in different types of biological samples, e.g., serum, plasma and sputum [4,11,52,53,54,55,56,57,58,59,60,61,62,63,64,65,66,67,68,69,70,71,72,73,74,75,76,77] (Table 1). Results were highly heterogeneous, even in studies using similar biological material. However, all of the works reported an alteration of circulating miRNA expression in cancer patients with respect to healthy donors or individuals with other non malignant lung diseases. Normalization of quantitative real-time polymerase chain reaction (qRT-PCR) data was performed in different ways, some authors using total RNA [11], others opting for serum volume [55], specific miRNAs e.g., RNU6B [56,58,65] or miR-16 [59,62,63,66,68], or miRNA ratio [4,67]. Unexpectedly, miRNAs used as normalizers in some studies proved to be promising biomarkers in others. Bianchi *et al.* identified miR-197 and miR-24 as among the most stable and least variable miRNAs in serum and used them as normalizers [53]. Conversely, other authors reported that miR-24 was upregulated in the serum of NSCLC patients with respect to healthy donors [55,59] and that miR-197 was upregulated in the plasma of NSCLC cases [61]. It can thus be concluded that there are several biases in the different methodological approaches that can only be resolved by method standardization and confirmatory studies. The majority of published works considered panels of miRNAs and a number of single miRNAs were consistently found to be deregulated. miR-21, one of the most frequently deregulated, was at least twofold upregulated in numerous studies [59,62,65,66,68,71]. Heterogeneous results were also obtained using whole peripheral blood as starting material (Table 1). Although various miRNA signatures were identified as markers capable of distinguishing between NSCLC patients and healthy donors, different studies identified different promising miRNAs [72,73,74,75,76,77,78]. The most consistent results for miRNAs were obtained when sputum was used as starting material; in these studies, RNA U6 was frequently used as normalizer. In particular, miR-21 and miR-210 were found to be the most deregulated miRNAs in sputum of NSCLC patients [79,80,81,82,83,84] (Table 1).

## 6. Diagnostic Potential of Circulating miRNAs

The strong stability of miRNAs in biological samples suggests that they could potentially be used as diagnostic, prognostic and predictive biomarkers for different tumor types [24,85,86]. The availability of non-invasive biomarkers would be a valuable tool for screening programs and to monitor suspicious CT-detected nodules. Numerous studies have focused on identifying miRNAs that are capable of discriminating between NSCLC patients and healthy donors or individuals with non-neoplastic diseases, with sensitivity and specificity ranging from 60% to 100% (Table 1) [4,51,53,56,60,61,62,63,65,66,67,68,71,73,74,75,76,77,79,80,81,82,83,84,87]. In the majority of these studies, initial miRNA profiling was performed using “large spectrum” methodologies, e.g., chip array or low-density array. The most promising miRNAs were then validated with qRT-PCR methods and almost all studies identified a panel of miRNAs rather than a single miRNA that showed good diagnostic accuracy in discriminating between cancer patients and healthy individuals (Table 1).

One of the first studies to focus on the diagnostic accuracy of miRNA expression in serum was carried out by Bianchi *et al.* [53] who evaluated a panel of 361 miRNAs in patients from the COSMOS trial [88]. The authors showed that a 34-miRNA signature was capable of discriminating between healthy individuals and patients with adenocarcinoma (ADC) or squamous cell carcinoma (SCC). In particular, an AUC ROC value of 0.89, with 71% sensitivity and 90% specificity, was obtained in the testing set in which 30 healthy donors were compared with 22 ADC and 12 SCC patients. Moreover, the diagnostic accuracy increased and reached an AUC ROC of 0.94 when a comparison was made between healthy donors and only SCC patients. Using a more restricted panel of 10 miRNAs, Chen *et al.* [55] obtained higher diagnostic accuracy. In their validating set comprising 110 healthy donors and 200 NSCLC patients, they obtained an AUC ROC value of 0.972. Similar results were obtained by Hennessey *et al.* [52] using only two miRNAs (miR-15b and miR-27b). They began their study by evaluating 328 miRNAs in a group of 20 healthy donors and 16 NSCLC patients, but subsequently restricted the analysis to 26 miRNAs in 75 healthy donors and 55 NSCLC cases. Although different miRNA pairs showed good diagnostic accuracy, the best pair was miR-15b and miR-27b, which showed absolute specificity and 84% sensitivity. Numerous other studies have also analyzed the diagnostic potential of miRNAs in plasma (Table 1). Recently, Sozzi *et al.* hypothesized the potential for using miRNAs in screening programs [4]. The authors considered patients enrolled onto the MILD clinical trial [89] and identified a miRNA signature classifier (MSC) which, when used together with low dose computed tomography (LDCT), increased screening sensitivity to 98%, with a false-positive rate of only 35%. Furthermore, the false-positive rate of LDCT was reduced more than fivefold when patients double-positive for MSC and LDCT were taken into consideration.

**Table 1:** Studies on circulating miRNAs as biomarkers for lung cancer.

Ref.	Case Series	Biological Material	Methodologies	Normalization	Promising miRNAs	Diagnostic Potential
[11]	75 HD 152 NSCLC	Serum	Solexa deep sequencing/qRT-PCR	Total RNA	miR-25, miR-223	-
[52]	75 HD 55 NSCLC	Serum	qRT-PCR	-	miR-15b miR-27b pair	Sens = 100% Spec = 84% PPV = 82% NPV = 100% AUC = 0.98
[53]	39 HD 25 ADC (Training set)	Serum	qRT-PCR	miR-197, miR-19b, miR-24, miR-146, miR-15b, miR-19a	Panel of 34 miRNAs	Sens = 69% Spec = 84% AUC = 0.92
	30 HD 34 NSCLC (Testing set)					Sens = 71% Spec = 90% AUC = 0.89
[54]	6 HD 8 NSCLC	Serum	qRT-PCR	-	miR-16, miR-518a-5p, miR-574-5p, miR-593, miR-663, miR-718, miR-1228, miR-1972, miR-2114	-
[55]	110 HD 200 NSCLC	Serum	qRT-PCR	Serum volume	miR-222, miR-199a-5p, miR-320, miR-20a, miR-24, miR-223, miR-25, miR-152, miR-145, miR-221	AUC = 0.97
[56]	11 HD 11 NSCLC (Training set)	Serum	qRT-PCR	RNU6B, miR-39	miR-1254, miR-574-5p	Sens = 82% Spec = 77% AUC = 0.77
	31 HD 22 NSCLC (Validating set)					Sens = 73% Spec = 71% AUC = 0.75
[57]	40 HD 40 NSCLC (ADC)	Serum	qRT-PCR	-	miR-30c, miR-616, miR-146b-3p, miR-566, miR-550, miR-939, miR339-5p, miR-656	AUC of different miRNAs: miR-30c = 0.74, miR-616 = 0.81, miR-146b-3p = 0.71, miR-566 = 0.79, miR-550 = 0.72, miR-939 = 0.82, miR-339-5p = 0.60, miR-656 = 0.60
[58]	30 HD 20 benign diseases 97 NSCLC	Serum	qRT-PCR	RNU6B, miR-1233	miR-361-3p, miR-625	AUC miR-361-3p = 0.86, AUC miR-625 = 0.77
[59]	50 HD 82 NSCLC pre-surgery 10 NSCLC post surgery	Serum	qRT-PCR	miR-16	miR-21, miR-205, miR-30d, miR-24	AUC miR-21 = 0.70, AUC miR-205 = 0.81, AUC miR-30d = 0.76, AUC miR-24 = 0.86
[60]	110 HD 193 NSCLC	Serum	qRT-PCR	-	miR-125b	Sens = 78% Spec = 66% AUC = 0.79
[61]	68 HD 74 lung cancer	Plasma	qRT-PCR	-	miR-155, miR-197, miR-182	Sens = 81.3% Spec = 86.8% AUC = 0.90
[63]	29 HD 58 NSCLC	Plasma	qRT-PCR	miR-16	miR-21, miR-126, miR-210, miR-486-5p	Sens = 86.2% Spec = 96.6% AUC = 0.93
[64]	48 HD 78 NSCLC	Plasma vesicles	qRT-PCR	miR-142-3p, miR-30b	let-7f, miR-20b, miR-30e-3p	-
[62]	80 BSN 76 MSN	Plasma	qRT-PCR	miR-16	miR-21, miR-210, miR-486-5p	Sens = 76.3% Spec = 85% AUC = 0.86
[65]	60 HD 62 NSCLC	Plasma	qRT-PCR	RNU6B	miR-21, miR-145, miR-155	Sens = 69.4% Spec = 78.3% AUC = 0.85
	32 HD 34 NSCLC (Validation set)					Sens = 76.5% Spec = 81.3% AUC = 0.87
[66]	46 HD 54 NSCLC	Plasma	qRT-PCR	miR-16	miR-21, miR-486	Sens = 87% Spec = 86.5% AUC = 0.90
		EBC	qRT-PCR	miR-16		Sens = 60% Spec = 71.1% AUC = 0.68
[67]	10 HD 16 NSCLC	Plasma	qRT-PCR	miRNA ratio	16-miRNA ratio	Sens = 75% Spec = 100% AUC = 0.88
[68]	30 HD 63 NSCLC	Plasma	qRT-PCR	miR-16	miR-21	Sens = 76.2% Spec = 70% AUC = 0.78
[69]	20 HD 62 NSCLC	Serum	qRT-PCR	RNU6B	miR-126, miR-183	-
[70]	220 HD 220 NSCLC	Serum and plasma	qRT-PCR	*C. elegans* miRs (cel-miR-54, cel-miR-238)	miR-146b, miR-221, let-7a, miR-155, miR-17-5p, miR-27a, miR-106a, miR-29c	AUC = 0.60
[4]	870 disease-free individuals at screening 69 NSCLC	Plasma	qRT-PCR	miRNA ratio	miRNA signature classifier	Sens = 87% Spec = 81%
[71]	38 HD 36 NSCLC	Plasma	qRT-PCR and digital PCR	-	miR-21-5p, miR-335-3p	Sens = 71.8% Spec = 80.6% AUC = 0.86
[72]	30 HD 35 NSCLC	Whole blood	qRT-PCR	RNU6B	let-7a	AUC = 0.95
[73]	91 NMLD 137 NSCLC (Training set)	Whole blood	Array		29-miRNA signature	Sens = 91% Spec = 80% AUC = 0.92
	17 NMLD 38 NSCLC (Validation set)	Whole blood			29-miRNA signature	Sens = 76% Spec = 82%
[74]	19 HD 17 NSCLC	Whole blood	Array	-	24-miRNA signature	Sens = 92.5% Spec = 98.1%
[75]	19 HD 24 COPD 28 NSCLC	Whole blood	Array	-	250-miRNA signature	NSCLC vs COPD Sens = 92% Spec = 89%
[76]	23 HD 22 NSCLC	Whole blood	Array and qRT-PCR	-	miR-190b, miR-630, miR-942, miR-1284	TSP method: Sens = 91% Spec = 100% SVM method: Sens = 88% Spec = 89%
[77]	24 HD 86 NSCLC	Whole blood	qRT-PCR	RNU38B, RNU58A	miR-328	Sens = 70% Spec = 83% AUC = 0.82
[78]	26 HD 64 NSCLC	Whole blood	qRT-PCR	RNU6B	miR-143, miR-150	AUC miR-143 = 0.89 AUC miR-150 = 0.83
[87]	10 HD 10 granuloma 10 ADC	Exosome	qRT-PCR	let-7a	miR-378a, miR-379, miR-139-5p, miR-200b-5p (screening test) miR-151a-5p, miR-30a-3p, miR-200b-5p, miR-629, miR-100, miR154-3p (diagnostic test)	Screening test Sens = 97.5% Spec = 72% AUC = 0.91 Diagnostic test Sens = 96% Spec = 60% AUC = 0.76
[79]	17 HD 23 NSCLC	Sputum	qRT-PCR	RNU6B	miR-21	Sens = 69.7% Spec = 100% AUC = 0.90
[80]	36 HD 36 ADC [80] (Training set)	Sputum	qRT-PCR	RNU6B	miR-21, miR-486, miR-375, miR-200b	Sens = 80.6% Spec = 91.7% AUC = 0.90
	58 HD 64 ADC (Validation set)					Sens = 70.3% Spec = 80.0% AUC = 0.83
[81]	48 HD 48 SCC [80] (Training set)	Sputum	qRT-PCR	RNU6B	miR-205, miR-210, miR-708	Sens = 73% Spec = 96% AUC = 0.87
	55 HD 67 SCC (Validation set)					Sens = 72% Spec = 95%
[82]	6 HD 24 NSCLC	Sputum	qRT-PCR	RNU6B	miR-21, miR-155, miR-210, miR-143, miR-372	Sens = 83.3% Spec = 100%
[83]	68 HD 66 NSCLC (Training set)	Sputum	qRT-PCR	RNU6B	miR-31, miR-210	Sens = 65.2% Spec = 89.7% AUC = 0.83
	73 HD 64 NSCLC (Validation set)					Sens = 64.1% Spec = 89.2%
[84]	40 HD 35 NSCLC	Sputum	qRT-PCR	RNU6B	miR-31, miR-210	Sens = 65.7% Spec = 85.00% AUC = 0.86

HD: healthy donor; NSCLC: non small cell lung cancer; BSN: benign solitary nodule; MSN: malignant solitary nodule; EBC: exhalate breath condensate; NMLD: non malignant lung disease; ADC: adenocarcinoma; COPD: chronic obstructive pulmonary disease; TSP: top scoring pairs; SVM: support vector machines; SCC: squamous cell carcinoma; qRT-PCR: quatitative real-time polymerase chain reaction; Sens: sensitivity; Spec: specificity.

Other studies have aimed to identify miRNAs in whole peripheral blood that are capable of distinguishing between NSCLC patients and healthy donors. This would not only permit miRNAs released by the tumor to be evaluated, but also those expressed by blood cells, which could reflect specific inflammatory or immune-modulatory processes that occur during carcinogenesis. One of the first studies in this area was conducted by Showe *et al.* [73] who identified a 29-miRNA signature capable of discriminating between 137 patients with NSCLC and 91 patients with non-malignant lung diseases. Sensitivity and specificity were 91% and 80% in the training set and 76% and 82% in the validating set, respectively. Subsequent studies identified other miRNA signatures showing high diagnostic accuracy [74,75].

Research has also focused on the diagnostic potential of miRNAs analyzed in sputum. As before, some studies initially evaluated a large panel of miRNAs by microarray and then validated the most promising miRNAs by RT-PCR [80,81]. Yu *et al.* [80] identified a panel of four miRNAs that discriminated between healthy donors and patients with ADC. They also demonstrated that three other miRNAs were capable of distinguishing healthy donors from patients with SCC [81]. More recently, the same group showed the high diagnostic accuracy of only two miRNAs in discriminating between healthy donors and patients with either ADC or SCC [82,83] (Table 1).

## 7. Conclusions and Future Prospects

Robustness and reproducibility are key requisites for the clinical implementation of biomarkers. Although qRT-PCR based multiplex assays for the detection of miRNAs are straightforward and robust, they are still hampered by a lack of agreement about the normalization approach and the use of an adequate internal control. There is still no general consensus in the literature regarding the best method to use and a standardized protocol would help to guarantee the reproducibility of results on different biological samples (blood, serum or plasma and sputum) obtained by non-invasive methods. Moreover, although the majority of studies conducted to date confirmed their own results through a training and validating set, almost all were based on small case series which reduced the diagnostic power of the biomarkers.

In conclusion, although the future of miRNAs as non-invasive diagnostic markers for the early detection of NSCLC looks promising, a number of important issues, *i.e.*, protocol standardization, choice of normalization factors, and definition of the best biological samples to use for this type of analysis, need to be addressed.
